# Tetra­carbonyl­bis(η^5^-cyclo­penta­dienyl)bis[(dec-9-en-1-yl)diphenyl­phosphine]dimolybdenum(0)(*Mo*—*Mo*) tetra­hydro­furan disolvate

**DOI:** 10.1107/S1600536809046327

**Published:** 2009-11-11

**Authors:** Ginger Shultz, Lev N. Zakharov, David R Tyler

**Affiliations:** a Department of Chemistry, 1253 University of Oregon, Eugene, Oregon 97403-1253, USA

## Abstract

The asymmetric unit of the title compound, [Mo_2_(η^5^-C_5_H_5_)_2_(C_22_H_29_P)_2_(CO)_4_]·2C_4_H_8_O, contains two half-mol­ecules of the organometallic species and two solvent mol­ecules. Both organometallic mol­ecules are completed by crystallographic inversion symmetry, yielding dimeric units with Mo—Mo single-bond lengths of 3.2703 (6) and 3.2548 (6) Å. Each Mo atom is also coordinated by an η^5^-cyclo­pentdienyl ligand, two carbonyl ligands, and a (dec-9-en-1-yl)diphenyl­phosphine ligand.

## Related literature

For related literature from our group in this area, see: Chen *et al.* (2004 ▶); Daglen *et al.* (2007 ▶); For other compounds containing Mo—Mo single bonds, see: Wilson & Shoemaker (1957 ▶); Shultz *et al.* (2008 ▶).
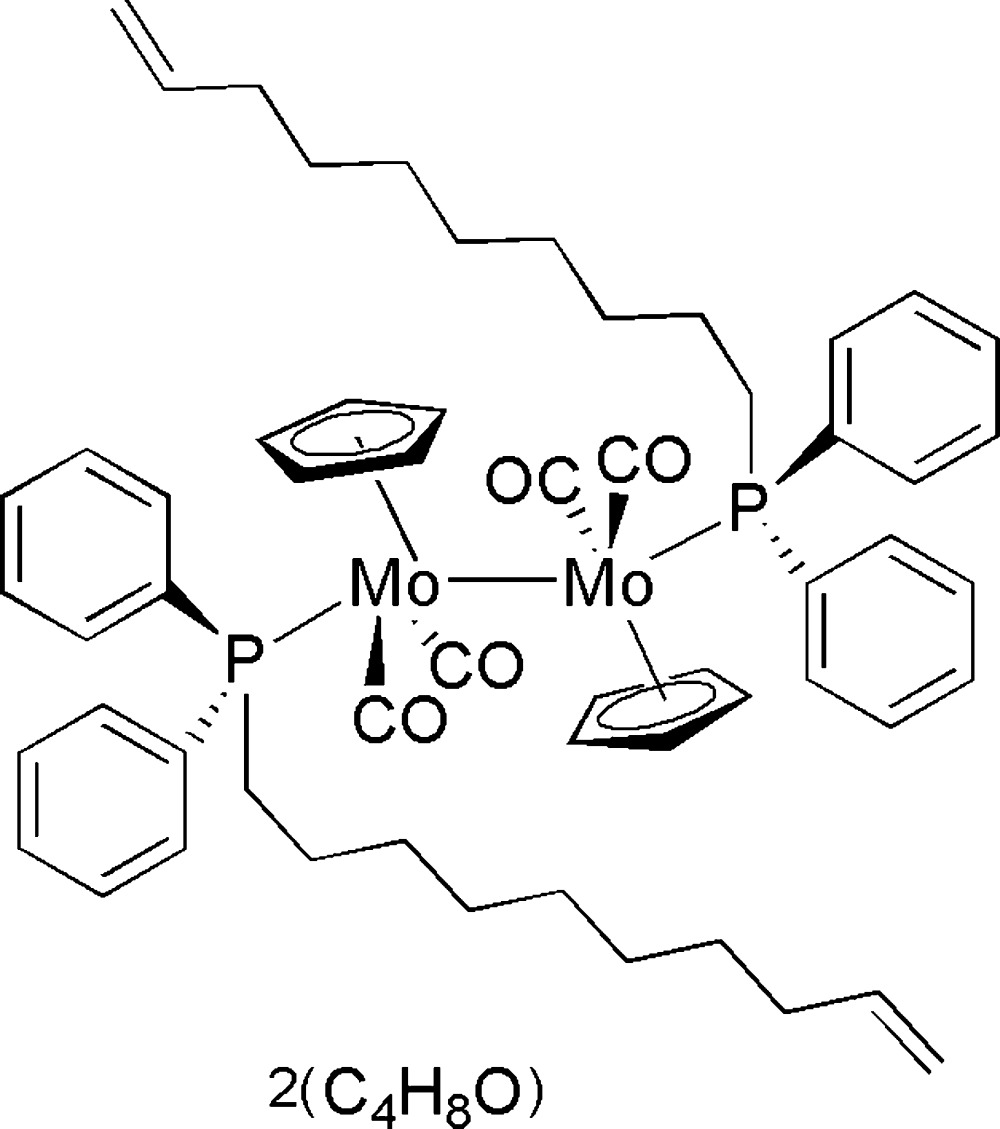


## Experimental

### 

#### Crystal data

[Mo_2_(C_5_H_5_)_2_(C_22_H_29_P)_2_(CO)_4_]·2C_4_H_8_O
*M* *_r_* = 1227.16
Triclinic, 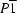

*a* = 11.2755 (4) Å
*b* = 15.5660 (5) Å
*c* = 18.8566 (6) Å
α = 109.166 (1)°
β = 98.008 (1)°
γ = 101.323 (1)°
*V* = 2990.20 (17) Å^3^
*Z* = 2
Mo *K*α radiation
μ = 0.52 mm^−1^
*T* = 173 K
0.21 × 0.12 × 0.02 mm

#### Data collection

Bruker APEX CCD area-detector diffractometer
Absorption correction: multi-scan (*SADABS*; Bruker, 2000 ▶) *T*_min_ = 0.898, *T*_max_ = 0.990
33790 measured reflections
12955 independent reflections
10237 reflections with *I* > 2σ(*I*)
*R*_int_ = 0.036

#### Refinement

*R*[*F* ^2^ > 2σ(*F* ^2^)] = 0.047
*wR*(*F* ^2^) = 0.121
*S* = 1.05
12955 reflections
686 parameters
H-atom parameters constrained
Δρ_max_ = 0.73 e Å^−3^
Δρ_min_ = −0.68 e Å^−3^

### 

Data collection: *SMART* (Bruker, 2000 ▶); cell refinement: *SAINT* (Bruker, 2000 ▶); data reduction: *SAINT*; program(s) used to solve structure: *SHELXTL* (Sheldrick, 2008 ▶); program(s) used to refine structure: *SHELXTL*; molecular graphics: *SHELXTL*; software used to prepare material for publication: *SHELXTL*.

## Supplementary Material

Crystal structure: contains datablocks I, global. DOI: 10.1107/S1600536809046327/hb5140sup1.cif

Structure factors: contains datablocks I. DOI: 10.1107/S1600536809046327/hb5140Isup2.hkl

Additional supplementary materials:  crystallographic information; 3D view; checkCIF report

## Figures and Tables

**Table 1:** Selected bond lengths (Å)

Mo1—P1	2.4337 (10)
Mo2—P2	2.4191 (9)

**Fig. 1.**
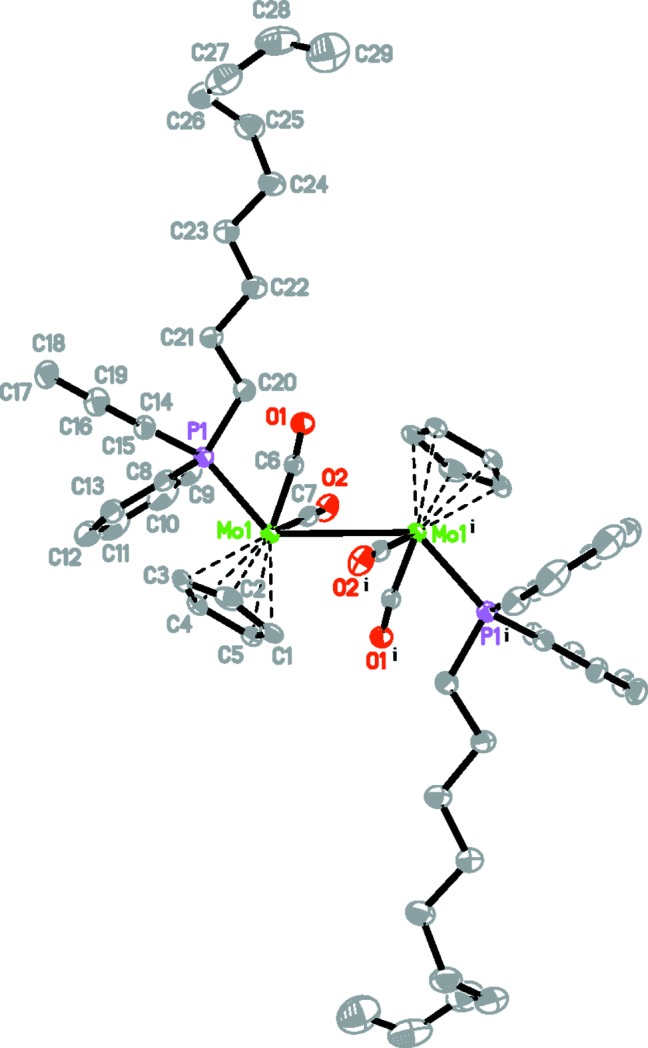
The structure of the Mo1 molecule of (I) with 50% probability displacement ellipsoids Symmetry code (i): -x,-y,-z].

## supplementary crystallographic information

### Comment

In an on-going mechanistic study in our lab we have examined the effects of external parameters, such as mechanical stress (Chen *et al.*, 2004) and temperature (Daglen *et al.*, 2007), on the rate and onset of polymer degradation using polymers with metal-metal bonds in the main chain. Such polymers are synthesized from functionalized dimeric metal carbonyl complexes. Recently, we examined ADMET, Acyclic Diene Metathesis Polymerization, as a method for preparing polymers with metal-metal bonds, using phosphine-substituted dimeric molybdenum complexes as precursors (Shultz *et al.*, 2008). Specifically the title complex was synthesized for polymerization by ADMET and purification of this complex yielded single crystals.

The structure of complex 1 reported here consists of two centrosymmetrical units [(η^5^-C_5_H_5_)Mo(CO)_2_(Ph_2_P(CH_2_)_8_CH=CH_2_)]_2_ (where Cp is cyclopentadienyl) and two solvent (C_4_H_8_O) molecules. In each unit the Mo atom is coordinated by a Cp moiety, two carbonyl groups and a Ph_2_P(CH_2_)_8_CH=CH_2_ ligand in a piano-stool fashion and bonded by a Mo—Mo bond as well (Fig. 1). The Mo—Mo bond lengths in 1, 3.2703 (6) and 3.2548 (6) Å, are within the range of single Mo—Mo bond lengths observed in other relevant dimeric molybdenum complexes such as [(η^5^-C_5_H_5_)Mo(CO)_2_]_2_ (Wilson & Shoemaker, 1957) and [MoCp(CO)_2_(Ph_2_P(CH_2_)_6_CH=CH_2_)]_2_ (Shultz *et al.*, 2008).

### Experimental

The synthesis of the title compound was carried out under rigorous light and air free conditions. A solution of Cp_2_Mo_2_(CO)_4_ (408.15 mg, 0.94 mmol) in CH_2_Cl_2_ (10 ml) was treated with a solution of Ph_2_P(CH_2_)_8_CH=CH_2_ (481.84 mg, 1.88 mmol) in CH_2_Cl_2_ (10 ml) by adding dropwise while stirring. Red plates of (I) were obtained by cooling a solution of the complex in THF/CH_2_Cl_2_/hexanes.

### Refinement

The H atoms were positioned geometrically and refined with riding or rigid group models: C—H = 1.00 (Cp-ring), 0.95 (Ph-rings) and 0.99 (CH_2_ groups) Å; *U*_iso_(H) = 1.2*U*_eq_(C).

### Crystal data

**Table:**

[Mo_2_(C_5_H_5_)_2_(C_22_H_29_P)_2_(CO)_4_]·2C_4_H_8_O	*Z* = 2
*M**_r_* = 1227.16	*F*(000) = 1284
Triclinic, *P*1	*D*_x_ = 1.363 Mg m^−^^3^
Hall symbol: -P 1	Mo *K*α radiation, λ = 0.71073 Å
*a* = 11.2755 (4) Å	Cell parameters from 6981 reflections
*b* = 15.5660 (5) Å	θ = 2.3–24.5°
*c* = 18.8566 (6) Å	µ = 0.52 mm^−^^1^
α = 109.166 (1)°	*T* = 173 K
β = 98.008 (1)°	Plate, red
γ = 101.323 (1)°	0.21 × 0.12 × 0.02 mm
*V* = 2990.20 (17) Å^3^	

### Data collection

**Table:**

Bruker APEX CCD area-detector diffractometer	12955 independent reflections
Radiation source: fine-focus sealed tube	10237 reflections with *I* > 2σ(*I*)
graphite	*R*_int_ = 0.036
φ and ω scans	θ_max_ = 27.0°, θ_min_ = 1.2°
Absorption correction: multi-scan (*SADABS*; Bruker, 2000)	*h* = −14→14
*T*_min_ = 0.898, *T*_max_ = 0.990	*k* = −19→19
33790 measured reflections	*l* = −24→24

### Refinement

**Table:**

Refinement on *F*^2^	Primary atom site location: structure-invariant direct methods
Least-squares matrix: full	Secondary atom site location: difference Fourier map
*R*[*F*^2^ > 2σ(*F*^2^)] = 0.047	Hydrogen site location: inferred from neighbouring sites
*wR*(*F*^2^) = 0.121	H-atom parameters constrained
*S* = 1.05	*w* = 1/[σ^2^(*F*_o_^2^) + (0.0507*P*)^2^ + 2.7213*P*] where *P* = (*F*_o_^2^ + 2*F*_c_^2^)/3
12955 reflections	(Δ/σ)_max_ = 0.001
686 parameters	Δρ_max_ = 0.73 e Å^−^^3^
0 restraints	Δρ_min_ = −0.68 e Å^−^^3^

### Special details

**Table:**

Geometry. All e.s.d.'s (except the e.s.d. in the dihedral angle between two l.s. planes)are estimated using the full covariance matrix. The cell e.s.d.'s are takeninto account individually in the estimation of e.s.d.'s in distances, anglesand torsion angles; correlations between e.s.d.'s in cell parameters are onlyused when they are defined by crystal symmetry. An approximate (isotropic)treatment of cell e.s.d.'s is used for estimating e.s.d.'s involving l.s. planes.
Refinement. Refinement of *F*^2^ against ALL reflections. The weighted *R*-factor *wR* and goodness of fit *S* are based on *F*^2^, conventional *R*-factors *R* are based on *F*, with *F* set to zero for negative *F*^2^. The threshold expression of *F*^2^ > σ(*F*^2^) is used only for calculating *R*-factors(gt) *etc*. and is not relevant to the choice of reflections for refinement. *R*-factors based on *F*^2^ are statistically about twice as large as those based on *F*, and *R*- factors based on ALL data will be even larger.

### Fractional atomic coordinates and isotropic or equivalent isotropic displacement parameters (Å^2^)

**Table:**

	*x*	*y*	*z*	*U*_iso_*/*U*_eq_	
Mo1	0.04404 (3)	0.54395 (2)	0.093859 (16)	0.02578 (8)	
P1	0.09180 (8)	0.70546 (6)	0.18453 (5)	0.0290 (2)	
O1	−0.1968 (2)	0.60007 (19)	0.04523 (15)	0.0399 (6)	
O2	0.2526 (2)	0.64265 (19)	0.03326 (16)	0.0458 (7)	
C1	0.0195 (4)	0.3859 (3)	0.0856 (2)	0.0397 (9)	
H1A	−0.0125	0.3288	0.0373	0.048*	
C2	−0.0525 (4)	0.4267 (3)	0.1359 (3)	0.0484 (11)	
H2A	−0.1437	0.4031	0.1303	0.058*	
C3	0.0289 (4)	0.5008 (3)	0.1992 (2)	0.0469 (11)	
H3A	0.0056	0.5380	0.2470	0.056*	
C4	0.1508 (4)	0.5050 (3)	0.1885 (2)	0.0414 (9)	
H4A	0.2284	0.5457	0.2272	0.050*	
C5	0.1446 (4)	0.4336 (3)	0.1179 (2)	0.0387 (9)	
H5A	0.2169	0.4160	0.0972	0.046*	
C6	−0.1050 (3)	0.5791 (2)	0.05908 (19)	0.0310 (8)	
C7	0.1711 (3)	0.6050 (2)	0.0525 (2)	0.0342 (8)	
C8	0.2490 (3)	0.7483 (3)	0.2454 (2)	0.0358 (8)	
C9	0.3483 (4)	0.7917 (3)	0.2219 (3)	0.0482 (10)	
H9A	0.3338	0.8052	0.1764	0.058*	
C10	0.4679 (4)	0.8150 (4)	0.2647 (3)	0.0682 (15)	
H10A	0.5351	0.8456	0.2490	0.082*	
C11	0.4899 (5)	0.7941 (4)	0.3301 (3)	0.0768 (18)	
H11A	0.5725	0.8085	0.3584	0.092*	
C12	0.3935 (5)	0.7526 (4)	0.3544 (3)	0.0648 (14)	
H12A	0.4092	0.7394	0.4000	0.078*	
C13	0.2728 (4)	0.7296 (3)	0.3127 (2)	0.0468 (10)	
H13A	0.2061	0.7010	0.3301	0.056*	
C14	−0.0038 (3)	0.7368 (3)	0.2558 (2)	0.0321 (8)	
C15	−0.1242 (3)	0.6820 (3)	0.2424 (2)	0.0370 (8)	
H15A	−0.1557	0.6278	0.1966	0.044*	
C16	−0.1987 (4)	0.7050 (3)	0.2947 (2)	0.0465 (10)	
H16A	−0.2806	0.6669	0.2847	0.056*	
C17	−0.1528 (4)	0.7838 (3)	0.3612 (2)	0.0512 (11)	
H17A	−0.2032	0.7995	0.3975	0.061*	
C18	−0.0355 (4)	0.8395 (3)	0.3754 (2)	0.0484 (10)	
H18A	−0.0053	0.8941	0.4211	0.058*	
C19	0.0400 (4)	0.8165 (3)	0.3232 (2)	0.0402 (9)	
H19A	0.1216	0.8552	0.3336	0.048*	
C20	0.0808 (4)	0.7893 (3)	0.1360 (2)	0.0384 (9)	
H20A	0.1410	0.7840	0.1022	0.046*	
H20B	−0.0028	0.7674	0.1018	0.046*	
C21	0.1017 (4)	0.8945 (3)	0.1826 (2)	0.0427 (9)	
H21A	0.0458	0.9028	0.2190	0.051*	
H21B	0.1883	0.9212	0.2127	0.051*	
C22	0.0760 (4)	0.9465 (3)	0.1282 (3)	0.0480 (10)	
H22A	0.1381	0.9429	0.0957	0.058*	
H22B	−0.0067	0.9135	0.0936	0.058*	
C23	0.0792 (4)	1.0484 (3)	0.1679 (3)	0.0509 (11)	
H23A	0.1638	1.0835	0.1989	0.061*	
H23B	0.0214	1.0533	0.2032	0.061*	
C24	0.0424 (5)	1.0919 (3)	0.1088 (3)	0.0571 (12)	
H24A	0.0990	1.0839	0.0728	0.069*	
H24B	−0.0423	1.0560	0.0785	0.069*	
C25	0.0446 (5)	1.1941 (3)	0.1412 (3)	0.0684 (14)	
H25A	0.0225	1.2147	0.0979	0.082*	
H25B	0.1304	1.2305	0.1689	0.082*	
C26	−0.0423 (6)	1.2185 (4)	0.1961 (4)	0.0849 (18)	
H26A	−0.0151	1.2039	0.2420	0.102*	
H26B	−0.0343	1.2872	0.2134	0.102*	
C27	−0.1782 (7)	1.1669 (4)	0.1614 (4)	0.093 (2)	
H27A	−0.1869	1.0986	0.1481	0.111*	
H27B	−0.2264	1.1868	0.2012	0.111*	
C28	−0.2335 (6)	1.1819 (5)	0.0925 (5)	0.098 (2)	
H28A	−0.2349	1.2449	0.0993	0.117*	
C29	−0.2788 (7)	1.1222 (6)	0.0261 (5)	0.123 (3)	
H29A	−0.2802	1.0580	0.0156	0.147*	
H29B	−0.3119	1.1411	−0.0138	0.147*	
Mo2	0.45151 (3)	0.43825 (2)	0.408367 (16)	0.02669 (9)	
P2	0.56343 (9)	0.39119 (7)	0.30767 (5)	0.0320 (2)	
O3	0.6040 (3)	0.63647 (19)	0.42382 (15)	0.0410 (6)	
O4	0.6224 (3)	0.33879 (19)	0.48002 (15)	0.0445 (7)	
C30	0.2469 (3)	0.4366 (3)	0.4266 (2)	0.0375 (9)	
H30A	0.2249	0.4754	0.4743	0.045*	
C31	0.2578 (3)	0.4615 (3)	0.3621 (2)	0.0359 (8)	
H31A	0.2438	0.5202	0.3555	0.043*	
C32	0.2811 (3)	0.3847 (3)	0.3056 (2)	0.0345 (8)	
H32A	0.2841	0.3790	0.2516	0.041*	
C33	0.2825 (3)	0.3122 (3)	0.3353 (2)	0.0369 (8)	
H33A	0.2862	0.2468	0.3058	0.044*	
C34	0.2619 (3)	0.3446 (3)	0.4103 (2)	0.0380 (9)	
H34A	0.2501	0.3066	0.4436	0.046*	
C35	0.5534 (3)	0.5630 (3)	0.42241 (19)	0.0321 (8)	
C36	0.5626 (3)	0.3807 (3)	0.4562 (2)	0.0338 (8)	
C37	0.5122 (4)	0.2638 (3)	0.2506 (2)	0.0400 (9)	
C38	0.5452 (4)	0.2006 (3)	0.2828 (3)	0.0498 (10)	
H38A	0.5983	0.2242	0.3326	0.060*	
C39	0.5025 (5)	0.1046 (3)	0.2441 (3)	0.0601 (12)	
H39A	0.5258	0.0627	0.2671	0.072*	
C40	0.4258 (5)	0.0696 (3)	0.1717 (3)	0.0648 (14)	
H40A	0.3977	0.0035	0.1445	0.078*	
C41	0.3899 (5)	0.1303 (4)	0.1389 (3)	0.0648 (13)	
H41A	0.3360	0.1060	0.0894	0.078*	
C42	0.4330 (4)	0.2275 (3)	0.1786 (2)	0.0506 (11)	
H42A	0.4077	0.2691	0.1560	0.061*	
C43	0.5537 (3)	0.4431 (3)	0.2330 (2)	0.0339 (8)	
C44	0.4886 (3)	0.5112 (3)	0.2358 (2)	0.0376 (9)	
H44A	0.4481	0.5311	0.2770	0.045*	
C45	0.4819 (4)	0.5503 (3)	0.1794 (2)	0.0433 (9)	
H45A	0.4364	0.5960	0.1818	0.052*	
C46	0.5419 (4)	0.5224 (3)	0.1198 (2)	0.0500 (11)	
H46A	0.5377	0.5490	0.0811	0.060*	
C47	0.6075 (4)	0.4562 (3)	0.1167 (2)	0.0507 (11)	
H47A	0.6489	0.4374	0.0759	0.061*	
C48	0.6136 (4)	0.4167 (3)	0.1725 (2)	0.0438 (10)	
H48A	0.6594	0.3709	0.1695	0.053*	
C49	0.7325 (3)	0.4054 (3)	0.3349 (2)	0.0412 (9)	
H49A	0.7481	0.3780	0.3747	0.049*	
H49B	0.7597	0.3687	0.2892	0.049*	
C50	0.8117 (3)	0.5060 (3)	0.3651 (2)	0.0393 (9)	
H50A	0.7835	0.5441	0.4097	0.047*	
H50B	0.8010	0.5330	0.3246	0.047*	
C51	0.9497 (4)	0.5115 (3)	0.3899 (2)	0.0470 (10)	
H51A	0.9596	0.4855	0.4311	0.056*	
H51B	0.9761	0.4713	0.3455	0.056*	
C52	1.0339 (4)	0.6096 (3)	0.4185 (2)	0.0510 (11)	
H52A	1.0187	0.6371	0.3787	0.061*	
H52B	1.1208	0.6050	0.4244	0.061*	
C53	1.0201 (5)	0.6769 (3)	0.4941 (2)	0.0567 (12)	
H53A	1.0220	0.6457	0.5321	0.068*	
H53B	0.9381	0.6904	0.4862	0.068*	
C54	1.1215 (4)	0.7710 (4)	0.5275 (3)	0.0605 (12)	
H54A	1.2036	0.7570	0.5319	0.073*	
H54B	1.1160	0.8038	0.4907	0.073*	
C55	1.1147 (6)	0.8347 (4)	0.6027 (3)	0.0864 (18)	
H55A	1.1250	0.8030	0.6400	0.104*	
H55B	1.0306	0.8452	0.5989	0.104*	
C56	1.2094 (5)	0.9304 (4)	0.6348 (3)	0.0793 (16)	
H56A	1.2919	0.9207	0.6279	0.095*	
H56B	1.1872	0.9684	0.6046	0.095*	
C57	1.2186 (7)	0.9840 (5)	0.7170 (5)	0.114 (2)	
H57A	1.2461	0.9548	0.7509	0.137*	
C58	1.1963 (6)	1.0619 (5)	0.7500 (5)	0.103 (2)	
H58A	1.1684	1.0960	0.7204	0.124*	
H58B	1.2074	1.0865	0.8044	0.124*	
O1S	1.2824 (4)	1.1169 (3)	0.9547 (3)	0.1031 (15)	
O2S	0.2211 (5)	0.0400 (4)	0.4745 (3)	0.1144 (16)	
C1S	1.4026 (6)	1.1116 (5)	0.9393 (5)	0.108 (2)	
H1SA	1.3957	1.0721	0.8849	0.130*	
H1SB	1.4461	1.0851	0.9730	0.130*	
C2S	1.4673 (8)	1.2091 (6)	0.9558 (7)	0.166 (4)	
H2SA	1.5225	1.2362	1.0077	0.199*	
H2SB	1.5184	1.2124	0.9177	0.199*	
C3S	1.3780 (7)	1.2588 (5)	0.9520 (6)	0.141 (4)	
H3SA	1.3555	1.2597	0.8996	0.170*	
H3SB	1.4089	1.3245	0.9892	0.170*	
C4S	1.2724 (7)	1.2088 (5)	0.9713 (5)	0.112 (3)	
H4SA	1.2731	1.2387	1.0265	0.135*	
H4SB	1.1941	1.2093	0.9405	0.135*	
C5S	0.3311 (7)	0.0087 (5)	0.4703 (4)	0.094 (2)	
H5SA	0.3108	−0.0608	0.4524	0.112*	
H5SB	0.3878	0.0349	0.5217	0.112*	
C6S	0.3910 (8)	0.0415 (6)	0.4155 (5)	0.134 (3)	
H6SA	0.4817	0.0667	0.4356	0.160*	
H6SB	0.3748	−0.0102	0.3649	0.160*	
C7S	0.3325 (8)	0.1159 (6)	0.4095 (6)	0.134 (3)	
H7SA	0.3840	0.1784	0.4455	0.161*	
H7SB	0.3215	0.1159	0.3565	0.161*	
C8S	0.2144 (8)	0.0944 (5)	0.4292 (5)	0.112 (2)	
H8SA	0.1938	0.1533	0.4578	0.134*	
H8SB	0.1487	0.0594	0.3818	0.134*	

### Atomic displacement parameters (Å^2^)

**Table:**

	*U*^11^	*U*^22^	*U*^33^	*U*^12^	*U*^13^	*U*^23^
Mo1	0.02704 (16)	0.02878 (16)	0.02517 (16)	0.00944 (12)	0.00681 (12)	0.01285 (12)
P1	0.0259 (5)	0.0319 (5)	0.0303 (5)	0.0083 (4)	0.0075 (4)	0.0119 (4)
O1	0.0352 (15)	0.0501 (16)	0.0402 (15)	0.0230 (13)	0.0103 (12)	0.0164 (13)
O2	0.0375 (16)	0.0449 (16)	0.0477 (17)	−0.0013 (12)	0.0208 (13)	0.0097 (13)
C1	0.052 (2)	0.032 (2)	0.041 (2)	0.0110 (17)	0.0059 (19)	0.0223 (17)
C2	0.051 (3)	0.051 (3)	0.063 (3)	0.016 (2)	0.024 (2)	0.042 (2)
C3	0.075 (3)	0.054 (3)	0.033 (2)	0.032 (2)	0.024 (2)	0.029 (2)
C4	0.054 (3)	0.045 (2)	0.031 (2)	0.0215 (19)	0.0014 (18)	0.0179 (18)
C5	0.049 (2)	0.041 (2)	0.035 (2)	0.0221 (18)	0.0083 (18)	0.0190 (17)
C6	0.036 (2)	0.0311 (18)	0.0272 (18)	0.0095 (15)	0.0096 (15)	0.0110 (15)
C7	0.036 (2)	0.0320 (19)	0.0325 (19)	0.0120 (16)	0.0086 (16)	0.0073 (16)
C8	0.0289 (19)	0.0342 (19)	0.036 (2)	0.0102 (15)	0.0033 (16)	0.0023 (16)
C9	0.031 (2)	0.045 (2)	0.056 (3)	0.0042 (18)	0.0104 (19)	0.006 (2)
C10	0.027 (2)	0.063 (3)	0.084 (4)	0.001 (2)	0.010 (2)	−0.006 (3)
C11	0.036 (3)	0.078 (4)	0.078 (4)	0.022 (3)	−0.016 (3)	−0.014 (3)
C12	0.058 (3)	0.070 (3)	0.050 (3)	0.033 (3)	−0.011 (2)	0.001 (2)
C13	0.042 (2)	0.048 (2)	0.042 (2)	0.0159 (19)	−0.0003 (19)	0.0067 (19)
C14	0.0303 (19)	0.038 (2)	0.0318 (19)	0.0126 (15)	0.0102 (15)	0.0134 (16)
C15	0.034 (2)	0.042 (2)	0.033 (2)	0.0099 (17)	0.0072 (16)	0.0115 (17)
C16	0.034 (2)	0.060 (3)	0.048 (2)	0.0125 (19)	0.0172 (19)	0.021 (2)
C17	0.047 (3)	0.072 (3)	0.043 (2)	0.028 (2)	0.022 (2)	0.021 (2)
C18	0.053 (3)	0.053 (3)	0.036 (2)	0.021 (2)	0.011 (2)	0.0071 (19)
C19	0.039 (2)	0.044 (2)	0.035 (2)	0.0117 (18)	0.0091 (17)	0.0103 (18)
C20	0.043 (2)	0.037 (2)	0.039 (2)	0.0092 (17)	0.0126 (18)	0.0180 (17)
C21	0.050 (2)	0.037 (2)	0.045 (2)	0.0163 (18)	0.0132 (19)	0.0157 (18)
C22	0.055 (3)	0.039 (2)	0.054 (3)	0.0134 (19)	0.012 (2)	0.020 (2)
C23	0.058 (3)	0.044 (2)	0.053 (3)	0.016 (2)	0.012 (2)	0.019 (2)
C24	0.069 (3)	0.051 (3)	0.062 (3)	0.021 (2)	0.018 (2)	0.030 (2)
C25	0.081 (4)	0.042 (3)	0.076 (4)	0.011 (2)	0.003 (3)	0.021 (3)
C26	0.124 (6)	0.059 (3)	0.082 (4)	0.042 (4)	0.032 (4)	0.025 (3)
C27	0.110 (6)	0.073 (4)	0.114 (5)	0.036 (4)	0.059 (5)	0.038 (4)
C28	0.073 (4)	0.058 (4)	0.153 (7)	0.017 (3)	0.017 (5)	0.030 (4)
C29	0.101 (6)	0.087 (5)	0.145 (8)	0.002 (4)	0.008 (5)	0.019 (5)
Mo2	0.02711 (16)	0.03345 (17)	0.02372 (16)	0.01178 (13)	0.01064 (12)	0.01154 (13)
P2	0.0309 (5)	0.0419 (5)	0.0283 (5)	0.0147 (4)	0.0135 (4)	0.0134 (4)
O3	0.0474 (16)	0.0424 (16)	0.0367 (15)	0.0090 (13)	0.0122 (12)	0.0193 (13)
O4	0.0522 (17)	0.0517 (17)	0.0413 (16)	0.0313 (14)	0.0126 (13)	0.0207 (13)
C30	0.0276 (19)	0.052 (2)	0.033 (2)	0.0126 (17)	0.0131 (16)	0.0122 (18)
C31	0.0265 (18)	0.046 (2)	0.038 (2)	0.0133 (16)	0.0075 (16)	0.0163 (17)
C32	0.0277 (18)	0.046 (2)	0.0279 (18)	0.0082 (16)	0.0044 (15)	0.0122 (16)
C33	0.032 (2)	0.038 (2)	0.035 (2)	0.0054 (16)	0.0081 (16)	0.0088 (17)
C34	0.0289 (19)	0.049 (2)	0.038 (2)	0.0052 (17)	0.0108 (16)	0.0203 (18)
C35	0.035 (2)	0.043 (2)	0.0249 (18)	0.0181 (17)	0.0115 (15)	0.0145 (16)
C36	0.037 (2)	0.037 (2)	0.0283 (18)	0.0103 (16)	0.0130 (16)	0.0104 (16)
C37	0.039 (2)	0.043 (2)	0.042 (2)	0.0165 (18)	0.0207 (18)	0.0120 (18)
C38	0.055 (3)	0.046 (2)	0.053 (3)	0.022 (2)	0.017 (2)	0.016 (2)
C39	0.062 (3)	0.049 (3)	0.070 (3)	0.021 (2)	0.019 (3)	0.018 (2)
C40	0.064 (3)	0.045 (3)	0.078 (4)	0.015 (2)	0.023 (3)	0.010 (3)
C41	0.065 (3)	0.061 (3)	0.054 (3)	0.009 (3)	0.012 (2)	0.007 (2)
C42	0.053 (3)	0.050 (3)	0.044 (2)	0.013 (2)	0.012 (2)	0.011 (2)
C43	0.0316 (19)	0.045 (2)	0.0259 (18)	0.0083 (16)	0.0109 (15)	0.0133 (16)
C44	0.037 (2)	0.047 (2)	0.0292 (19)	0.0091 (17)	0.0119 (16)	0.0142 (17)
C45	0.043 (2)	0.049 (2)	0.039 (2)	0.0077 (19)	0.0082 (18)	0.0195 (19)
C46	0.056 (3)	0.061 (3)	0.034 (2)	0.004 (2)	0.011 (2)	0.024 (2)
C47	0.054 (3)	0.064 (3)	0.036 (2)	0.010 (2)	0.025 (2)	0.017 (2)
C48	0.045 (2)	0.053 (2)	0.037 (2)	0.0144 (19)	0.0208 (19)	0.0156 (19)
C49	0.037 (2)	0.059 (3)	0.038 (2)	0.0244 (19)	0.0189 (18)	0.0216 (19)
C50	0.036 (2)	0.055 (2)	0.036 (2)	0.0182 (18)	0.0130 (17)	0.0222 (19)
C51	0.043 (2)	0.063 (3)	0.043 (2)	0.023 (2)	0.0136 (19)	0.022 (2)
C52	0.038 (2)	0.069 (3)	0.047 (3)	0.019 (2)	0.014 (2)	0.018 (2)
C53	0.067 (3)	0.066 (3)	0.043 (3)	0.020 (3)	0.015 (2)	0.025 (2)
C54	0.053 (3)	0.074 (3)	0.050 (3)	0.020 (3)	0.005 (2)	0.018 (2)
C55	0.093 (5)	0.071 (4)	0.082 (4)	0.012 (3)	0.030 (4)	0.012 (3)
C56	0.077 (4)	0.084 (4)	0.061 (3)	0.007 (3)	0.008 (3)	0.016 (3)
C57	0.110 (6)	0.087 (5)	0.134 (7)	0.017 (5)	0.042 (5)	0.026 (5)
C58	0.090 (5)	0.094 (5)	0.117 (6)	0.027 (4)	0.014 (4)	0.030 (5)
O1S	0.073 (3)	0.095 (3)	0.155 (4)	0.027 (2)	0.030 (3)	0.057 (3)
O2S	0.117 (4)	0.119 (4)	0.142 (4)	0.047 (3)	0.050 (3)	0.073 (4)
C1S	0.083 (5)	0.091 (5)	0.164 (7)	0.035 (4)	0.044 (5)	0.050 (5)
C2S	0.100 (6)	0.112 (7)	0.315 (14)	0.019 (5)	0.089 (8)	0.106 (8)
C3S	0.072 (5)	0.074 (5)	0.270 (12)	0.011 (4)	−0.010 (6)	0.076 (6)
C4S	0.103 (6)	0.083 (5)	0.161 (7)	0.045 (4)	0.014 (5)	0.050 (5)
C5S	0.104 (5)	0.074 (4)	0.085 (5)	0.023 (4)	−0.008 (4)	0.020 (4)
C6S	0.160 (8)	0.172 (8)	0.161 (8)	0.113 (7)	0.099 (7)	0.112 (7)
C7S	0.168 (8)	0.134 (7)	0.182 (9)	0.082 (6)	0.095 (7)	0.113 (7)
C8S	0.141 (7)	0.107 (6)	0.127 (6)	0.071 (5)	0.049 (5)	0.064 (5)

### Geometric parameters (Å, °)

**Table:**

Mo1—C7	1.956 (4)	C30—C31	1.406 (5)
Mo1—C6	1.961 (4)	C30—C34	1.413 (5)
Mo1—C3	2.313 (4)	C30—H30A	1.0000
Mo1—C4	2.323 (3)	C31—C32	1.414 (5)
Mo1—C2	2.356 (4)	C31—H31A	1.0000
Mo1—C5	2.360 (4)	C32—C33	1.416 (5)
Mo1—C1	2.371 (4)	C32—H32A	1.0000
Mo1—P1	2.4337 (10)	C33—C34	1.405 (5)
P1—C8	1.835 (4)	C33—H33A	1.0000
P1—C20	1.838 (4)	C34—H34A	1.0000
P1—C14	1.840 (4)	C37—C42	1.385 (6)
O1—C6	1.165 (4)	C37—C38	1.397 (6)
O2—C7	1.158 (4)	C38—C39	1.379 (6)
C1—C5	1.401 (5)	C38—H38A	0.9500
C1—C2	1.409 (6)	C39—C40	1.380 (7)
C1—H1A	1.0000	C39—H39A	0.9500
C2—C3	1.404 (6)	C40—C41	1.380 (7)
C2—H2A	1.0000	C40—H40A	0.9500
C3—C4	1.409 (6)	C41—C42	1.397 (6)
C3—H3A	1.0000	C41—H41A	0.9500
C4—C5	1.408 (5)	C42—H42A	0.9500
C4—H4A	1.0000	C43—C48	1.395 (5)
C5—H5A	1.0000	C43—C44	1.394 (5)
C8—C9	1.393 (5)	C44—C45	1.388 (5)
C8—C13	1.396 (5)	C44—H44A	0.9500
C9—C10	1.384 (6)	C45—C46	1.385 (6)
C9—H9A	0.9500	C45—H45A	0.9500
C10—C11	1.377 (8)	C46—C47	1.372 (6)
C10—H10A	0.9500	C46—H46A	0.9500
C11—C12	1.366 (8)	C47—C48	1.382 (6)
C11—H11A	0.9500	C47—H47A	0.9500
C12—C13	1.387 (6)	C48—H48A	0.9500
C12—H12A	0.9500	C49—C50	1.516 (6)
C13—H13A	0.9500	C49—H49A	0.9900
C14—C15	1.394 (5)	C49—H49B	0.9900
C14—C19	1.395 (5)	C50—C51	1.537 (5)
C15—C16	1.387 (5)	C50—H50A	0.9900
C15—H15A	0.9500	C50—H50B	0.9900
C16—C17	1.381 (6)	C51—C52	1.507 (6)
C16—H16A	0.9500	C51—H51A	0.9900
C17—C18	1.369 (6)	C51—H51B	0.9900
C17—H17A	0.9500	C52—C53	1.515 (6)
C18—C19	1.394 (5)	C52—H52A	0.9900
C18—H18A	0.9500	C52—H52B	0.9900
C19—H19A	0.9500	C53—C54	1.544 (7)
C20—C21	1.534 (5)	C53—H53A	0.9900
C20—H20A	0.9900	C53—H53B	0.9900
C20—H20B	0.9900	C54—C55	1.462 (7)
C21—C22	1.531 (5)	C54—H54A	0.9900
C21—H21A	0.9900	C54—H54B	0.9900
C21—H21B	0.9900	C55—C56	1.526 (8)
C22—C23	1.506 (6)	C55—H55A	0.9900
C22—H22A	0.9900	C55—H55B	0.9900
C22—H22B	0.9900	C56—C57	1.477 (9)
C23—C24	1.532 (6)	C56—H56A	0.9900
C23—H23A	0.9900	C56—H56B	0.9900
C23—H23B	0.9900	C57—C58	1.259 (9)
C24—C25	1.499 (6)	C57—H57A	0.9500
C24—H24A	0.9900	C58—H58A	0.9500
C24—H24B	0.9900	C58—H58B	0.9500
C25—C26	1.532 (8)	O1S—C4S	1.391 (7)
C25—H25A	0.9900	O1S—C1S	1.437 (7)
C25—H25B	0.9900	O2S—C8S	1.392 (7)
C26—C27	1.521 (9)	O2S—C5S	1.422 (7)
C26—H26A	0.9900	C1S—C2S	1.457 (9)
C26—H26B	0.9900	C1S—H1SA	0.9900
C27—C28	1.471 (9)	C1S—H1SB	0.9900
C27—H27A	0.9900	C2S—C3S	1.394 (10)
C27—H27B	0.9900	C2S—H2SA	0.9900
C28—C29	1.250 (9)	C2S—H2SB	0.9900
C28—H28A	0.9500	C3S—C4S	1.449 (10)
C29—H29A	0.9500	C3S—H3SA	0.9900
C29—H29B	0.9500	C3S—H3SB	0.9900
Mo2—C36	1.960 (4)	C4S—H4SA	0.9900
Mo2—C35	1.965 (4)	C4S—H4SB	0.9900
Mo2—C33	2.325 (4)	C5S—C6S	1.480 (9)
Mo2—C32	2.327 (3)	C5S—H5SA	0.9900
Mo2—C34	2.351 (4)	C5S—H5SB	0.9900
Mo2—C30	2.375 (3)	C6S—C7S	1.467 (9)
Mo2—C31	2.378 (3)	C6S—H6SA	0.9900
Mo2—P2	2.4191 (9)	C6S—H6SB	0.9900
P2—C43	1.839 (4)	C7S—C8S	1.438 (10)
P2—C37	1.844 (4)	C7S—H7SA	0.9900
P2—C49	1.853 (4)	C7S—H7SB	0.9900
O3—C35	1.164 (4)	C8S—H8SA	0.9900
O4—C36	1.168 (4)	C8S—H8SB	0.9900
			
C7—Mo1—C6	103.92 (14)	C34—Mo2—P2	123.22 (10)
C7—Mo1—C3	139.05 (16)	C30—Mo2—P2	141.12 (9)
C6—Mo1—C3	111.19 (15)	C31—Mo2—P2	112.24 (9)
C7—Mo1—C4	105.76 (15)	C43—P2—C37	102.50 (17)
C6—Mo1—C4	146.03 (14)	C43—P2—C49	101.89 (17)
C3—Mo1—C4	35.38 (14)	C37—P2—C49	99.87 (18)
C7—Mo1—C2	155.25 (15)	C43—P2—Mo2	118.30 (12)
C6—Mo1—C2	97.77 (15)	C37—P2—Mo2	112.64 (12)
C3—Mo1—C2	34.98 (15)	C49—P2—Mo2	118.88 (13)
C4—Mo1—C2	58.31 (15)	C31—C30—C34	108.8 (3)
C7—Mo1—C5	97.95 (14)	C31—C30—Mo2	72.9 (2)
C6—Mo1—C5	151.64 (15)	C34—C30—Mo2	71.7 (2)
C3—Mo1—C5	58.30 (14)	C31—C30—H30A	125.5
C4—Mo1—C5	34.99 (13)	C34—C30—H30A	125.5
C2—Mo1—C5	57.84 (14)	Mo2—C30—H30A	125.5
C7—Mo1—C1	121.87 (14)	C30—C31—C32	107.3 (3)
C6—Mo1—C1	117.22 (14)	C30—C31—Mo2	72.7 (2)
C3—Mo1—C1	57.91 (14)	C32—C31—Mo2	70.6 (2)
C4—Mo1—C1	57.82 (14)	C30—C31—H31A	126.2
C2—Mo1—C1	34.67 (14)	C32—C31—H31A	126.2
C5—Mo1—C1	34.44 (13)	Mo2—C31—H31A	126.2
C7—Mo1—P1	79.76 (10)	C31—C32—C33	108.3 (3)
C6—Mo1—P1	80.28 (10)	C31—C32—Mo2	74.5 (2)
C3—Mo1—P1	85.92 (11)	C33—C32—Mo2	72.2 (2)
C4—Mo1—P1	89.06 (10)	C31—C32—H32A	125.6
C2—Mo1—P1	116.03 (12)	C33—C32—H32A	125.6
C5—Mo1—P1	121.76 (10)	Mo2—C32—H32A	125.6
C1—Mo1—P1	143.12 (10)	C34—C33—C32	107.9 (3)
C8—P1—C20	105.18 (18)	C34—C33—Mo2	73.5 (2)
C8—P1—C14	102.12 (17)	C32—C33—Mo2	72.4 (2)
C20—P1—C14	102.22 (17)	C34—C33—H33A	125.8
C8—P1—Mo1	114.01 (12)	C32—C33—H33A	125.8
C20—P1—Mo1	112.27 (13)	Mo2—C33—H33A	125.8
C14—P1—Mo1	119.39 (12)	C33—C34—C30	107.7 (3)
C5—C1—C2	108.6 (4)	C33—C34—Mo2	71.5 (2)
C5—C1—Mo1	72.4 (2)	C30—C34—Mo2	73.5 (2)
C2—C1—Mo1	72.1 (2)	C33—C34—H34A	125.9
C5—C1—H1A	125.6	C30—C34—H34A	125.9
C2—C1—H1A	125.6	Mo2—C34—H34A	125.9
Mo1—C1—H1A	125.6	O3—C35—Mo2	172.3 (3)
C3—C2—C1	107.5 (4)	O4—C36—Mo2	174.0 (3)
C3—C2—Mo1	70.8 (2)	C42—C37—C38	118.2 (4)
C1—C2—Mo1	73.2 (2)	C42—C37—P2	122.1 (3)
C3—C2—H2A	126.1	C38—C37—P2	119.5 (3)
C1—C2—H2A	126.1	C39—C38—C37	121.4 (4)
Mo1—C2—H2A	126.1	C39—C38—H38A	119.3
C2—C3—C4	108.3 (4)	C37—C38—H38A	119.3
C2—C3—Mo1	74.2 (2)	C38—C39—C40	119.7 (5)
C4—C3—Mo1	72.7 (2)	C38—C39—H39A	120.2
C2—C3—H3A	125.6	C40—C39—H39A	120.2
C4—C3—H3A	125.6	C39—C40—C41	120.2 (5)
Mo1—C3—H3A	125.6	C39—C40—H40A	119.9
C5—C4—C3	107.8 (4)	C41—C40—H40A	119.9
C5—C4—Mo1	73.9 (2)	C40—C41—C42	119.8 (5)
C3—C4—Mo1	71.9 (2)	C40—C41—H41A	120.1
C5—C4—H4A	125.9	C42—C41—H41A	120.1
C3—C4—H4A	125.9	C37—C42—C41	120.7 (4)
Mo1—C4—H4A	125.9	C37—C42—H42A	119.7
C1—C5—C4	107.8 (4)	C41—C42—H42A	119.7
C1—C5—Mo1	73.2 (2)	C48—C43—C44	117.8 (3)
C4—C5—Mo1	71.1 (2)	C48—C43—P2	120.8 (3)
C1—C5—H5A	126.0	C44—C43—P2	121.4 (3)
C4—C5—H5A	126.0	C45—C44—C43	121.1 (3)
Mo1—C5—H5A	126.0	C45—C44—H44A	119.5
O1—C6—Mo1	173.2 (3)	C43—C44—H44A	119.5
O2—C7—Mo1	174.7 (3)	C46—C45—C44	119.8 (4)
C9—C8—C13	118.7 (4)	C46—C45—H45A	120.1
C9—C8—P1	121.0 (3)	C44—C45—H45A	120.1
C13—C8—P1	120.0 (3)	C47—C46—C45	119.8 (4)
C10—C9—C8	120.3 (5)	C47—C46—H46A	120.1
C10—C9—H9A	119.9	C45—C46—H46A	120.1
C8—C9—H9A	119.9	C46—C47—C48	120.6 (4)
C11—C10—C9	120.2 (5)	C46—C47—H47A	119.7
C11—C10—H10A	119.9	C48—C47—H47A	119.7
C9—C10—H10A	119.9	C47—C48—C43	120.9 (4)
C12—C11—C10	120.3 (4)	C47—C48—H48A	119.6
C12—C11—H11A	119.9	C43—C48—H48A	119.6
C10—C11—H11A	119.9	C50—C49—P2	115.2 (3)
C11—C12—C13	120.4 (5)	C50—C49—H49A	108.5
C11—C12—H12A	119.8	P2—C49—H49A	108.5
C13—C12—H12A	119.8	C50—C49—H49B	108.5
C12—C13—C8	120.2 (4)	P2—C49—H49B	108.5
C12—C13—H13A	119.9	H49A—C49—H49B	107.5
C8—C13—H13A	119.9	C49—C50—C51	111.6 (3)
C15—C14—C19	118.2 (3)	C49—C50—H50A	109.3
C15—C14—P1	120.2 (3)	C51—C50—H50A	109.3
C19—C14—P1	121.6 (3)	C49—C50—H50B	109.3
C16—C15—C14	121.2 (4)	C51—C50—H50B	109.3
C16—C15—H15A	119.4	H50A—C50—H50B	108.0
C14—C15—H15A	119.4	C52—C51—C50	114.2 (3)
C17—C16—C15	119.3 (4)	C52—C51—H51A	108.7
C17—C16—H16A	120.3	C50—C51—H51A	108.7
C15—C16—H16A	120.3	C52—C51—H51B	108.7
C18—C17—C16	120.6 (4)	C50—C51—H51B	108.7
C18—C17—H17A	119.7	H51A—C51—H51B	107.6
C16—C17—H17A	119.7	C51—C52—C53	115.9 (4)
C17—C18—C19	120.2 (4)	C51—C52—H52A	108.3
C17—C18—H18A	119.9	C53—C52—H52A	108.3
C19—C18—H18A	119.9	C51—C52—H52B	108.3
C18—C19—C14	120.3 (4)	C53—C52—H52B	108.3
C18—C19—H19A	119.9	H52A—C52—H52B	107.4
C14—C19—H19A	119.9	C52—C53—C54	113.9 (4)
C21—C20—P1	120.8 (3)	C52—C53—H53A	108.8
C21—C20—H20A	107.1	C54—C53—H53A	108.8
P1—C20—H20A	107.1	C52—C53—H53B	108.8
C21—C20—H20B	107.1	C54—C53—H53B	108.8
P1—C20—H20B	107.1	H53A—C53—H53B	107.7
H20A—C20—H20B	106.8	C55—C54—C53	114.9 (4)
C22—C21—C20	109.8 (3)	C55—C54—H54A	108.5
C22—C21—H21A	109.7	C53—C54—H54A	108.5
C20—C21—H21A	109.7	C55—C54—H54B	108.5
C22—C21—H21B	109.7	C53—C54—H54B	108.5
C20—C21—H21B	109.7	H54A—C54—H54B	107.5
H21A—C21—H21B	108.2	C54—C55—C56	115.8 (5)
C23—C22—C21	114.6 (4)	C54—C55—H55A	108.3
C23—C22—H22A	108.6	C56—C55—H55A	108.3
C21—C22—H22A	108.6	C54—C55—H55B	108.3
C23—C22—H22B	108.6	C56—C55—H55B	108.3
C21—C22—H22B	108.6	H55A—C55—H55B	107.4
H22A—C22—H22B	107.6	C57—C56—C55	114.2 (5)
C22—C23—C24	110.6 (4)	C57—C56—H56A	108.7
C22—C23—H23A	109.5	C55—C56—H56A	108.7
C24—C23—H23A	109.5	C57—C56—H56B	108.7
C22—C23—H23B	109.5	C55—C56—H56B	108.7
C24—C23—H23B	109.5	H56A—C56—H56B	107.6
H23A—C23—H23B	108.1	C58—C57—C56	131.2 (9)
C25—C24—C23	115.8 (4)	C58—C57—H57A	114.4
C25—C24—H24A	108.3	C56—C57—H57A	114.4
C23—C24—H24A	108.3	C57—C58—H58A	120.0
C25—C24—H24B	108.3	C57—C58—H58B	120.0
C23—C24—H24B	108.3	H58A—C58—H58B	120.0
H24A—C24—H24B	107.4	C4S—O1S—C1S	109.4 (5)
C24—C25—C26	115.3 (4)	C8S—O2S—C5S	108.2 (5)
C24—C25—H25A	108.5	O1S—C1S—C2S	104.0 (6)
C26—C25—H25A	108.5	O1S—C1S—H1SA	111.0
C24—C25—H25B	108.5	C2S—C1S—H1SA	111.0
C26—C25—H25B	108.5	O1S—C1S—H1SB	111.0
H25A—C25—H25B	107.5	C2S—C1S—H1SB	111.0
C27—C26—C25	114.5 (5)	H1SA—C1S—H1SB	109.0
C27—C26—H26A	108.6	C3S—C2S—C1S	107.6 (7)
C25—C26—H26A	108.6	C3S—C2S—H2SA	110.2
C27—C26—H26B	108.6	C1S—C2S—H2SA	110.2
C25—C26—H26B	108.6	C3S—C2S—H2SB	110.2
H26A—C26—H26B	107.6	C1S—C2S—H2SB	110.2
C28—C27—C26	115.4 (5)	H2SA—C2S—H2SB	108.5
C28—C27—H27A	108.4	C2S—C3S—C4S	105.0 (6)
C26—C27—H27A	108.4	C2S—C3S—H3SA	110.7
C28—C27—H27B	108.4	C4S—C3S—H3SA	110.7
C26—C27—H27B	108.4	C2S—C3S—H3SB	110.7
H27A—C27—H27B	107.5	C4S—C3S—H3SB	110.7
C29—C28—C27	128.2 (7)	H3SA—C3S—H3SB	108.8
C29—C28—H28A	115.9	O1S—C4S—C3S	106.8 (6)
C27—C28—H28A	115.9	O1S—C4S—H4SA	110.4
C28—C29—H29A	120.0	C3S—C4S—H4SA	110.4
C28—C29—H29B	120.0	O1S—C4S—H4SB	110.4
H29A—C29—H29B	120.0	C3S—C4S—H4SB	110.4
C36—Mo2—C35	105.41 (15)	H4SA—C4S—H4SB	108.6
C36—Mo2—C33	103.58 (14)	O2S—C5S—C6S	107.9 (5)
C35—Mo2—C33	147.55 (14)	O2S—C5S—H5SA	110.1
C36—Mo2—C32	136.06 (14)	C6S—C5S—H5SA	110.1
C35—Mo2—C32	112.24 (14)	O2S—C5S—H5SB	110.1
C33—Mo2—C32	35.45 (13)	C6S—C5S—H5SB	110.1
C36—Mo2—C34	98.15 (14)	H5SA—C5S—H5SB	108.4
C35—Mo2—C34	148.90 (14)	C7S—C6S—C5S	103.4 (6)
C33—Mo2—C34	34.96 (12)	C7S—C6S—H6SA	111.1
C32—Mo2—C34	58.37 (13)	C5S—C6S—H6SA	111.1
C36—Mo2—C30	124.24 (14)	C7S—C6S—H6SB	111.1
C35—Mo2—C30	114.15 (14)	C5S—C6S—H6SB	111.1
C33—Mo2—C30	57.91 (13)	H6SA—C6S—H6SB	109.1
C32—Mo2—C30	57.76 (13)	C8S—C7S—C6S	105.7 (6)
C34—Mo2—C30	34.78 (13)	C8S—C7S—H7SA	110.6
C36—Mo2—C31	156.08 (14)	C6S—C7S—H7SA	110.6
C35—Mo2—C31	96.64 (14)	C8S—C7S—H7SB	110.6
C33—Mo2—C31	58.36 (13)	C6S—C7S—H7SB	110.6
C32—Mo2—C31	34.95 (12)	H7SA—C7S—H7SB	108.7
C34—Mo2—C31	57.97 (13)	O2S—C8S—C7S	108.5 (6)
C30—Mo2—C31	34.42 (12)	O2S—C8S—H8SA	110.0
C36—Mo2—P2	80.78 (10)	C7S—C8S—H8SA	110.0
C35—Mo2—P2	81.04 (10)	O2S—C8S—H8SB	110.0
C33—Mo2—P2	89.57 (9)	C7S—C8S—H8SB	110.0
C32—Mo2—P2	83.46 (9)	H8SA—C8S—H8SB	108.4
			
C7—Mo1—P1—C8	−66.73 (18)	C30—Mo2—P2—C43	60.8 (2)
C6—Mo1—P1—C8	−172.92 (17)	C31—Mo2—P2—C43	36.68 (17)
C3—Mo1—P1—C8	74.75 (18)	C36—Mo2—P2—C37	76.37 (18)
C4—Mo1—P1—C8	39.47 (18)	C35—Mo2—P2—C37	−176.29 (17)
C2—Mo1—P1—C8	93.12 (19)	C33—Mo2—P2—C37	−27.47 (17)
C5—Mo1—P1—C8	26.34 (19)	C32—Mo2—P2—C37	−62.47 (17)
C1—Mo1—P1—C8	64.1 (2)	C34—Mo2—P2—C37	−17.38 (19)
C7—Mo1—P1—C20	52.75 (18)	C30—Mo2—P2—C37	−58.6 (2)
C6—Mo1—P1—C20	−53.44 (17)	C31—Mo2—P2—C37	−82.73 (18)
C3—Mo1—P1—C20	−165.77 (18)	C36—Mo2—P2—C49	−39.88 (19)
C4—Mo1—P1—C20	158.96 (17)	C35—Mo2—P2—C49	67.47 (18)
C2—Mo1—P1—C20	−147.39 (18)	C33—Mo2—P2—C49	−143.71 (18)
C5—Mo1—P1—C20	145.83 (18)	C32—Mo2—P2—C49	−178.71 (18)
C1—Mo1—P1—C20	−176.5 (2)	C34—Mo2—P2—C49	−133.63 (19)
C7—Mo1—P1—C14	172.30 (17)	C30—Mo2—P2—C49	−174.9 (2)
C6—Mo1—P1—C14	66.11 (16)	C31—Mo2—P2—C49	161.03 (18)
C3—Mo1—P1—C14	−46.22 (17)	C36—Mo2—C30—C31	−164.4 (2)
C4—Mo1—P1—C14	−81.49 (17)	C35—Mo2—C30—C31	64.5 (3)
C2—Mo1—P1—C14	−27.84 (18)	C33—Mo2—C30—C31	−79.6 (2)
C5—Mo1—P1—C14	−94.62 (18)	C32—Mo2—C30—C31	−37.5 (2)
C1—Mo1—P1—C14	−56.9 (2)	C34—Mo2—C30—C31	−117.2 (3)
C7—Mo1—C1—C5	51.8 (3)	P2—Mo2—C30—C31	−42.0 (3)
C6—Mo1—C1—C5	−178.5 (2)	C36—Mo2—C30—C34	−47.2 (3)
C3—Mo1—C1—C5	−79.5 (3)	C35—Mo2—C30—C34	−178.3 (2)
C4—Mo1—C1—C5	−37.4 (2)	C33—Mo2—C30—C34	37.5 (2)
C2—Mo1—C1—C5	−117.0 (4)	C32—Mo2—C30—C34	79.7 (2)
P1—Mo1—C1—C5	−66.9 (3)	C31—Mo2—C30—C34	117.2 (3)
C7—Mo1—C1—C2	168.8 (2)	P2—Mo2—C30—C34	75.2 (3)
C6—Mo1—C1—C2	−61.6 (3)	C34—C30—C31—C32	−0.7 (4)
C3—Mo1—C1—C2	37.5 (3)	Mo2—C30—C31—C32	62.5 (2)
C4—Mo1—C1—C2	79.5 (3)	C34—C30—C31—Mo2	−63.1 (3)
C5—Mo1—C1—C2	117.0 (4)	C36—Mo2—C31—C30	33.4 (4)
P1—Mo1—C1—C2	50.1 (3)	C35—Mo2—C31—C30	−124.0 (2)
C5—C1—C2—C3	0.7 (4)	C33—Mo2—C31—C30	78.2 (2)
Mo1—C1—C2—C3	−63.0 (3)	C32—Mo2—C31—C30	116.1 (3)
C5—C1—C2—Mo1	63.6 (3)	C34—Mo2—C31—C30	36.8 (2)
C7—Mo1—C2—C3	92.7 (4)	P2—Mo2—C31—C30	153.0 (2)
C6—Mo1—C2—C3	−116.2 (3)	C36—Mo2—C31—C32	−82.8 (4)
C4—Mo1—C2—C3	37.9 (2)	C35—Mo2—C31—C32	119.9 (2)
C5—Mo1—C2—C3	79.4 (3)	C33—Mo2—C31—C32	−37.9 (2)
C1—Mo1—C2—C3	115.9 (4)	C34—Mo2—C31—C32	−79.4 (2)
P1—Mo1—C2—C3	−33.3 (3)	C30—Mo2—C31—C32	−116.1 (3)
C7—Mo1—C2—C1	−23.2 (5)	P2—Mo2—C31—C32	36.9 (2)
C6—Mo1—C2—C1	127.9 (3)	C30—C31—C32—C33	0.9 (4)
C3—Mo1—C2—C1	−115.9 (4)	Mo2—C31—C32—C33	64.8 (2)
C4—Mo1—C2—C1	−78.0 (3)	C30—C31—C32—Mo2	−63.9 (2)
C5—Mo1—C2—C1	−36.5 (2)	C36—Mo2—C32—C31	144.6 (2)
P1—Mo1—C2—C1	−149.2 (2)	C35—Mo2—C32—C31	−68.5 (2)
C1—C2—C3—C4	−0.7 (4)	C33—Mo2—C32—C31	115.5 (3)
Mo1—C2—C3—C4	−65.2 (3)	C34—Mo2—C32—C31	78.1 (2)
C1—C2—C3—Mo1	64.5 (3)	C30—Mo2—C32—C31	36.9 (2)
C7—Mo1—C3—C2	−140.3 (3)	P2—Mo2—C32—C31	−146.0 (2)
C6—Mo1—C3—C2	72.5 (3)	C36—Mo2—C32—C33	29.1 (3)
C4—Mo1—C3—C2	−115.5 (4)	C35—Mo2—C32—C33	176.0 (2)
C5—Mo1—C3—C2	−78.0 (3)	C34—Mo2—C32—C33	−37.4 (2)
C1—Mo1—C3—C2	−37.2 (2)	C30—Mo2—C32—C33	−78.6 (2)
P1—Mo1—C3—C2	150.4 (2)	C31—Mo2—C32—C33	−115.5 (3)
C7—Mo1—C3—C4	−24.9 (4)	P2—Mo2—C32—C33	98.5 (2)
C6—Mo1—C3—C4	−172.1 (2)	C31—C32—C33—C34	−0.8 (4)
C2—Mo1—C3—C4	115.5 (4)	Mo2—C32—C33—C34	65.5 (3)
C5—Mo1—C3—C4	37.5 (2)	C31—C32—C33—Mo2	−66.3 (3)
C1—Mo1—C3—C4	78.3 (3)	C36—Mo2—C33—C34	84.8 (2)
P1—Mo1—C3—C4	−94.1 (2)	C35—Mo2—C33—C34	−122.4 (3)
C2—C3—C4—C5	0.4 (4)	C32—Mo2—C33—C34	−115.5 (3)
Mo1—C3—C4—C5	−65.8 (3)	C30—Mo2—C33—C34	−37.3 (2)
C2—C3—C4—Mo1	66.2 (3)	C31—Mo2—C33—C34	−78.1 (2)
C7—Mo1—C4—C5	−81.2 (3)	P2—Mo2—C33—C34	165.2 (2)
C6—Mo1—C4—C5	128.7 (3)	C36—Mo2—C33—C32	−159.7 (2)
C3—Mo1—C4—C5	115.4 (4)	C35—Mo2—C33—C32	−6.9 (4)
C2—Mo1—C4—C5	78.0 (3)	C34—Mo2—C33—C32	115.5 (3)
C1—Mo1—C4—C5	36.8 (2)	C30—Mo2—C33—C32	78.2 (2)
P1—Mo1—C4—C5	−160.3 (2)	C31—Mo2—C33—C32	37.4 (2)
C7—Mo1—C4—C3	163.4 (2)	P2—Mo2—C33—C32	−79.3 (2)
C6—Mo1—C4—C3	13.3 (4)	C32—C33—C34—C30	0.4 (4)
C2—Mo1—C4—C3	−37.5 (2)	Mo2—C33—C34—C30	65.1 (3)
C5—Mo1—C4—C3	−115.4 (4)	C32—C33—C34—Mo2	−64.7 (3)
C1—Mo1—C4—C3	−78.6 (3)	C31—C30—C34—C33	0.1 (4)
P1—Mo1—C4—C3	84.3 (2)	Mo2—C30—C34—C33	−63.8 (3)
C2—C1—C5—C4	−0.4 (4)	C31—C30—C34—Mo2	63.9 (3)
Mo1—C1—C5—C4	63.1 (2)	C36—Mo2—C34—C33	−102.1 (2)
C2—C1—C5—Mo1	−63.4 (3)	C35—Mo2—C34—C33	118.7 (3)
C3—C4—C5—C1	0.0 (4)	C32—Mo2—C34—C33	37.9 (2)
Mo1—C4—C5—C1	−64.5 (2)	C30—Mo2—C34—C33	115.7 (3)
C3—C4—C5—Mo1	64.4 (3)	C31—Mo2—C34—C33	79.3 (2)
C7—Mo1—C5—C1	−137.6 (2)	P2—Mo2—C34—C33	−17.8 (3)
C6—Mo1—C5—C1	2.7 (4)	C36—Mo2—C34—C30	142.2 (2)
C3—Mo1—C5—C1	78.3 (3)	C35—Mo2—C34—C30	3.0 (4)
C4—Mo1—C5—C1	116.2 (4)	C33—Mo2—C34—C30	−115.7 (3)
C2—Mo1—C5—C1	36.8 (2)	C32—Mo2—C34—C30	−77.8 (2)
P1—Mo1—C5—C1	139.5 (2)	C31—Mo2—C34—C30	−36.4 (2)
C7—Mo1—C5—C4	106.2 (3)	P2—Mo2—C34—C30	−133.51 (19)
C6—Mo1—C5—C4	−113.5 (3)	C36—Mo2—C35—O3	158 (2)
C3—Mo1—C5—C4	−37.9 (3)	C33—Mo2—C35—O3	6(2)
C2—Mo1—C5—C4	−79.4 (3)	C32—Mo2—C35—O3	1(2)
C1—Mo1—C5—C4	−116.2 (4)	C34—Mo2—C35—O3	−64 (2)
P1—Mo1—C5—C4	23.3 (3)	C30—Mo2—C35—O3	−62 (2)
C7—Mo1—C6—O1	−146 (3)	C31—Mo2—C35—O3	−31 (2)
C3—Mo1—C6—O1	12 (3)	P2—Mo2—C35—O3	80 (2)
C4—Mo1—C6—O1	4(3)	C35—Mo2—C36—O4	−152 (3)
C2—Mo1—C6—O1	46 (3)	C33—Mo2—C36—O4	14 (3)
C5—Mo1—C6—O1	74 (3)	C32—Mo2—C36—O4	−3(3)
C1—Mo1—C6—O1	76 (3)	C34—Mo2—C36—O4	49 (3)
P1—Mo1—C6—O1	−70 (3)	C30—Mo2—C36—O4	74 (3)
C6—Mo1—C7—O2	141 (3)	C31—Mo2—C36—O4	52 (3)
C3—Mo1—C7—O2	−8(4)	P2—Mo2—C36—O4	−74 (3)
C4—Mo1—C7—O2	−22 (3)	C43—P2—C37—C42	−26.9 (4)
C2—Mo1—C7—O2	−69 (3)	C49—P2—C37—C42	−131.6 (3)
C5—Mo1—C7—O2	−57 (3)	Mo2—P2—C37—C42	101.3 (3)
C1—Mo1—C7—O2	−84 (3)	C43—P2—C37—C38	158.5 (3)
P1—Mo1—C7—O2	64 (3)	C49—P2—C37—C38	53.9 (3)
C20—P1—C8—C9	−34.2 (4)	Mo2—P2—C37—C38	−73.3 (3)
C14—P1—C8—C9	−140.7 (3)	C42—C37—C38—C39	1.1 (6)
Mo1—P1—C8—C9	89.2 (3)	P2—C37—C38—C39	175.9 (3)
C20—P1—C8—C13	152.0 (3)	C37—C38—C39—C40	0.2 (7)
C14—P1—C8—C13	45.6 (3)	C38—C39—C40—C41	−1.3 (8)
Mo1—P1—C8—C13	−84.6 (3)	C39—C40—C41—C42	1.0 (8)
C13—C8—C9—C10	0.4 (6)	C38—C37—C42—C41	−1.4 (6)
P1—C8—C9—C10	−173.5 (3)	P2—C37—C42—C41	−176.1 (3)
C8—C9—C10—C11	1.1 (7)	C40—C41—C42—C37	0.4 (7)
C9—C10—C11—C12	−1.9 (8)	C37—P2—C43—C48	−53.8 (3)
C10—C11—C12—C13	1.2 (8)	C49—P2—C43—C48	49.3 (4)
C11—C12—C13—C8	0.3 (7)	Mo2—P2—C43—C48	−178.3 (3)
C9—C8—C13—C12	−1.1 (6)	C37—P2—C43—C44	127.6 (3)
P1—C8—C13—C12	172.9 (3)	C49—P2—C43—C44	−129.3 (3)
C8—P1—C14—C15	−149.5 (3)	Mo2—P2—C43—C44	3.0 (4)
C20—P1—C14—C15	101.8 (3)	C48—C43—C44—C45	1.1 (6)
Mo1—P1—C14—C15	−22.8 (3)	P2—C43—C44—C45	179.8 (3)
C8—P1—C14—C19	32.1 (3)	C43—C44—C45—C46	−0.8 (6)
C20—P1—C14—C19	−76.6 (3)	C44—C45—C46—C47	0.1 (6)
Mo1—P1—C14—C19	158.9 (3)	C45—C46—C47—C48	0.3 (7)
C19—C14—C15—C16	−0.5 (6)	C46—C47—C48—C43	0.0 (7)
P1—C14—C15—C16	−179.0 (3)	C44—C43—C48—C47	−0.7 (6)
C14—C15—C16—C17	0.0 (6)	P2—C43—C48—C47	−179.4 (3)
C15—C16—C17—C18	0.7 (7)	C43—P2—C49—C50	59.4 (3)
C16—C17—C18—C19	−1.0 (7)	C37—P2—C49—C50	164.6 (3)
C17—C18—C19—C14	0.4 (6)	Mo2—P2—C49—C50	−72.6 (3)
C15—C14—C19—C18	0.3 (6)	P2—C49—C50—C51	177.2 (3)
P1—C14—C19—C18	178.7 (3)	C49—C50—C51—C52	178.4 (3)
C8—P1—C20—C21	−58.2 (3)	C50—C51—C52—C53	67.8 (5)
C14—P1—C20—C21	48.1 (4)	C51—C52—C53—C54	170.8 (4)
Mo1—P1—C20—C21	177.3 (3)	C52—C53—C54—C55	−176.4 (5)
P1—C20—C21—C22	−175.1 (3)	C53—C54—C55—C56	−176.6 (5)
C20—C21—C22—C23	173.3 (4)	C54—C55—C56—C57	−167.0 (6)
C21—C22—C23—C24	−175.5 (4)	C55—C56—C57—C58	−116.9 (9)
C22—C23—C24—C25	−178.8 (4)	C4S—O1S—C1S—C2S	5.7 (9)
C23—C24—C25—C26	−60.4 (6)	O1S—C1S—C2S—C3S	−20.8 (12)
C24—C25—C26—C27	−57.7 (7)	C1S—C2S—C3S—C4S	27.5 (12)
C25—C26—C27—C28	−58.8 (7)	C1S—O1S—C4S—C3S	10.8 (9)
C26—C27—C28—C29	118.3 (9)	C2S—C3S—C4S—O1S	−23.7 (11)
C36—Mo2—P2—C43	−164.23 (18)	C8S—O2S—C5S—C6S	−3.4 (8)
C35—Mo2—P2—C43	−56.88 (17)	O2S—C5S—C6S—C7S	17.6 (9)
C33—Mo2—P2—C43	91.94 (17)	C5S—C6S—C7S—C8S	−24.7 (10)
C32—Mo2—P2—C43	56.93 (17)	C5S—O2S—C8S—C7S	−12.7 (9)
C34—Mo2—P2—C43	102.02 (18)	C6S—C7S—C8S—O2S	23.9 (10)
